# Psychological Processes and Institutional Actors in the Sustainable Energy Transition: A Case-Study Analysis of a Local Community in Italy

**DOI:** 10.3389/fpsyg.2020.00980

**Published:** 2020-05-19

**Authors:** Lorenza Tiberio, Eugenio De Gregorio, Mehmet Efe Biresselioglu, Muhittin Hakan Demir, Angelo Panno, Giuseppe Carrus

**Affiliations:** ^1^Department of Education, Roma Tre University, Rome, Italy; ^2^Department of Education, University of Genoa, Genova, Italy; ^3^Sustainable Energy Division, İzmir University of Economics, İzmir, Turkey; ^4^Department of Logistics Management, İzmir University of Economics, İzmir, Turkey; ^5^Department of Human Science, European University of Rome, Rome, Italy

**Keywords:** energy choices, sustainable energy transition, formal social units, collective decision-making, qualitative research

## Abstract

This paper presents the results of a qualitative study exploring the role of formal social units in the sustainable energy transition process. A small community in North-east Italy was assumed as case study, in the context of a wider EU-funded research project aimed at understanding the individual and collective dimensions of energy-related choices. Starting from a general framework and integrated approach to explain sustainable energy transitions proposed by Steg et al. (2015), the main aim of our study was to identify the psychological and social factors that motivated the key actors to undertake an energy self-sufficiency path in the case-study area. Our analyses aimed at understanding the social, cultural, and socioeconomic dynamics in the energy transition process of the community. The study outlined how these factors contributed to foster collaborative actions between public decision makers, relevant stakeholders, civil society, and citizens. Results of a computer-supported qualitative content analysis using the software MAXQDA helped to shed light on the specific mechanisms and dynamics driving energy choices and energy-related behavior among the community. The implication for best practices and successful implementations of energy transition process in small communities emerging from this case study are discussed.

## Introduction

As reported by the European Climate Foundation, “in July 2009, the leaders of the European Union and the G8 announced an objective to reduce greenhouse gas emissions by at least 80% below 1990 levels by 2050” (European Climate Foundation, 2010, p. 3). Thus, 2050 is considered as a timely benchmark for policies addressing climate challenges. As energy-related issues represent a key feature in current global environmental change, this process also marks the era of the so-called energy transition (e.g., Solomon and Krishna, 2011). According to many international key players in this field (such as the IRENA-International Renewable Energy Agency)^1^, energy transition is merely a transition to a low carbon economy through the transformation of the energy sector, requiring a massive effort to replace fossil energy sources with renewable energy sources, although other authors have also underlined the importance of evolutionary and historical approaches to sustainable energy transitions (e.g., Verbong and Geels, 2007; Smil, 2010; Hirsh and Jones, 2014).

In the case of Europe, the pathway to energy transition has been planned through setting extremely ambitious goals and targets by the European Union and international agreements, and then transposing these goals into targets of the member and associate states. Although other paths, requiring more disruptive socio-technical transformations could also be possible, the first step in the EU official strategy has been to design measures and policies to attain these commonly set goals.

This phase of the energy transition is then primarily in the hands of formal social units, defined as institutions, policy makers and/or energy providers that have a central control over energy choice decisions. Formal social units include states, public authorities, municipalities and regional regulatory authorities.

The Energy Union, which is the major EU’s energy and climate policy strategy^2^, and the SET (Strategic Energy Technology) Plan^3^, which its technology pillar, are certainly among the most relevant measures and policies in this domain. The SET plan is developed as a decision support tool for Europe’s energy policy and as a framework to accelerate the development and deployment of cost-effective low carbon technologies. The SET plan aims to: (i) accelerate knowledge development, technology transfer and up-take; (ii) maintain EU industrial leadership on low-carbon energy technologies; (iii) foster science for transforming energy technologies to achieve the 2020 Energy and Climate Change goals (SET-Plan, 2008). However, a correct setting of overarching policies, goals, and targets does not itself guarantee a success for the energy transition, so that formal social units and decision makers also need to steer the operational facet of the energy transition by communicating and sharing it with the general public (e.g., Bain et al., 2019).

Hence, the competence and expertise of the formal social units has become even more critical in leading the energy transition process. In formal social units, the decision-making authority is, in general, given to top-level managers or elected leaders. Thus, the endeavor of highly motivated and enthusiastic people in charge of collective decisions is a very important asset for driving sustainable change in the energy domain, even for formal decision-making units where the institutional principles are in effect. Yet, public decision-makers also have important roles in acting as the interface for managing the communication with other stakeholders in all the phases of policymaking. Thus, investigating the point of view of formal decision makers is worth to understand the implementation of energy transition projects and the related decision-making processes, as well as the collective dimensions that influence the perception of economic, political and environmental factors affecting these decisions in collective settings (Biresselioglu et al., 2020).

Environmental psychological studies dedicated a great deal of theory and research in the past to understand the social and cognitive processes at the basis of ecological behaviors and sustainable choices in everyday life among “laypeople,” with significant achievements (e.g., Klöckner, 2013; Kaiser et al., 2014; Steg, 2016; Carrus et al., 2018; Evans et al., 2018; Panno et al., 2019). However, an interesting, yet still under investigated issue in this domain is related to the decision making and social cognitive processes of representatives of formal social units, such as, for example, members of elected bodies, public decision makers, administrative and company officers, civil society and public opinion leaders (e.g., Brondi et al., 2014, 2016; Sarrica et al., 2016a).

Thus, in this article, we explore the role that a formal social unit had in setting up an energy transition process in the specific case of a small rural community in northern Italy. The empirical data we present in this paper are part of the EU-funded research project “ECHOES,” aimed at understanding individual and collective energy-related choices and behaviors^4^. Our main research question here is related to identifying the psychological and social factors that affected the energy choices and energy related behavior of a formal social unit in the specific case study considered, and how these contributed to foster a collaborative action and manage the social, cultural, and socioeconomic dynamics in the energy transition path of the involved community.

## A General Framework on the Social Psychology of Sustainable Energy Choices

The ability to increase energy-related behavior largely depends on the behavioral choices of users, and on specific conditions that facilitate people’s willingness to accept energy policies and energy system changes. It is therefore essential to consider the social and psychological factors driving a successful implementation of an energy transition process at the individual, community and organizational level (e.g., Sarrica et al., 2014; Dumitru et al., 2016; Ruepert et al., 2016; Sarrica et al., 2016b, Sarrica et al., 2018a).

Some authors stressed the importance and strengths of community-based approaches to understand and steer the sustainable energy transition, particularly in rural, less-urbanized or less economically advantaged contexts (e.g., Sarrica et al., 2018b; Thomas et al., 2018). These studies start from a criticism to traditional, top–down and technology-based approaches to energy transition, rooted in neoclassical economics, and argue how these might not be ideal for implementing efficient distributed small-scale power generation needed by rural and remote communities (e.g., Thomas et al., 2018). Thus, community-based approaches underline the importance of considering the local understandings of energy and sustainability, and the dynamics of power at the local level in the shaping of community energy transitions (Sarrica et al., 2018b). More generally, a theoretical foundation for community-based approaches can be found in the multi-level perspective (MLP) to sustainable transitions (e.g., Geels, 2010), which identifies innovation niches, socio-technical regimes and exogenous technological landscapes as the core features driving any socio-technical transition, including sustainability-related ones. Recent developments have also criticized, and partly enlarged, this perspective, to incorporate issues of power and resistance dynamics for understanding the medium and long-term trajectory of the low-carbon societal shift (e.g., Geels, 2014).

**FIGURE 1:**
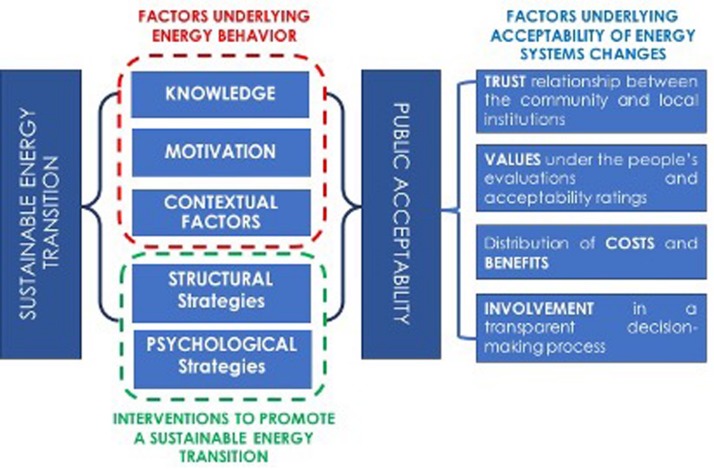
Graphic representation of the Sustainable Energy Transition model proposed by Steg et al. (2015), as revised by the authors.

From an environmental psychological perspective, Abrahamse et al. (2005); Steg (2008), and Steg et al. (2015) recently proposed an overarching framework to understand the human dimensions of sustainable energy transitions. In particular, Steg et al. (2015) argued that an integrated approach is essential in order to obtain a better understanding of the socio-psychological factors that may influence and encourage sustainable energy behaviors, as well as the acceptability of distinctive energy policies and energy system changes. This integrated approach is the result of an in-depth review of the contribution of social and environmental psychology. The authors considered on the one hand the role of three main factors on the adoption of sustainable energy behaviors, and on the other hand the importance and the characteristics of specific interventions aimed at promoting sustainable energy transitions: a graphic representation of the model proposed by Steg et al. (2015), and readapted to the proposed of this paper, is displayed in Figure 1.

Among the factors stimulating energy behavior, the review by Steg et al. (2015) highlights the specific role of knowledge, motivation and contextual factors. Knowledge, as such, is considered not sufficient to promote sustainable and pro-environmental energy behavior; empirical evidence shows that people often have limited knowledge of the causes and consequences of climate change; furthermore, individuals might often disregard the impact of human behavior on environmental global modifications. According to this proposal, even the inadequate or incorrect information on the characteristics of different types of alternative energy sources, and their effects on the environment, can influence the evaluation of the pros and cons of energy alternatives that are made available to the public. The limited influence of the knowledge factor is closely linked to personal and collective motivation. In order for people to adopt more sustainable energy behaviors and engage in actions aimed at environmental sustainability, individual and group motivation must be fostered and supported. People also need to feel able to engage in sustainable energy behaviors by perceiving that the individual costs of their behaviors are relatively minor in relation to the benefits that can be obtained. Cost and benefits involve instrumental, affective and social consequences of individual and collective choices related to sustainable energy use. Individual and collective motivational factors are also associated to the relation between environmental self-identity and the promotion of pro-environmental behaviors. Indeed, many authors argued about the identity dimension of pro-environmental and sustainable energy choices (e.g., Fritsche et al., 2018), and various studies recently reported how people who engage in sustainable energy behaviors strengthen their image as pro-environmental individuals (e.g., Masson et al., 2017; Carrus et al., 2019). Costly or uncommon pro-environmental behaviors have a greater influence on a person’s perception of the self as an individual interested to and involved in environmental sustainability issues. Among the motivational factors, Steg et al. (2015) also give emphasis to the role that hedonic, egoistic, biospheric and altruistic values have on evaluations, beliefs and actions, and how people consider and evaluate the individual and collective consequences of their sustainable energy behaviors.

An energy transition process also depends on how contextual factors promote or hinder the adoption of sustainable energy behaviors. Contextual factors refer to environmental, social and economic constraints that may influence individual and collective decision-making process by defining costs and benefits and thus influencing motivations. In terms of public acceptability of interventions and changes in energy systems, the authors also point out the role of the local context in which the opinions of citizens may also be determined on the basis of previous experiences or territorial characteristics. Hence, it is important to take into account the quality of the relations between the community and local institutions, for example in terms of trust, or the perception of being involved in a transparent decision-making process (e.g., Sarrica et al., 2018b). Thus, the role of values at the basis of people evaluations and acceptability ratings, and the perceived distribution of costs and benefits among individuals and the community, emerge as a crucial important factor to consider in a successful sustainable energy transition.

Reviewing which types of interventions are most effective for promoting a successful energy transition process, empirical evidence suggests that people might not be sufficiently motivated to act sustainably unless certain personal benefits are involved (e.g., Abrahamse et al., 2005). External incentives (e.g., financial aids, subsidies and tax discounts, governmental regulations) are among the most applied structural strategies to facilitate sustainable energy choices. However, previous research points to the unstable effect of incentives for consistent sustainable energy choices in the long run. In particular, the positive effects of financial incentives to promote ecological behavior often fades as soon as incentives are removed (Abrahamse et al., 2005). If the structural strategies appear to have an unstable effect in determining long-term behavioral changes, it is important to understand which strategies are more capable to strengthen the intrinsic motivation to engage in sustainable energy behaviors (see also Sousa Lourenço et al., 2016, for a more detailed review of behavioral interventions in many different policy domains).

Although adequately informed about energy problems, and energy savings options, and about the environmental and social problems caused by their behavior, people might not necessarily translate such knowledge and awareness baggage into actual and coherent energy choices. An alternative to the traditional information-based approach is the social influence approach, that is increasingly used to encourage pro-environmental behavior change (e.g., Schultz et al., 2015). Strategies such as block leaders’ approach, commitments, or social comparative feedback may act more effectively on individuals’ perception and the evaluation of the pros and cons of sustainable energy behavior.

Considering the current scientific debate on the human dimensions of the sustainable energy transition process, well summarized in the above referenced literature, it is arguable that this process requires fundamental and large-scale changes in human perceptions, preferences and behavior. Therefore, it is essential to understand how to motivate and enable people to actively contribute to a sustainable energy transition, not only from the perspective of the single individual or consumer, but also from the wider perspective of institutions, collective bodies and formal social units.

Starting from the general framework and integrated approach proposed by Steg et al. (2015), in this paper we use a case-study qualitative approach to explore the psychological and social factors affecting energy choices and energy related behaviors as reported by representative actors of a formal social unit, and how these factors might have contributed to successfully implement and sustain the energy transition path in a small rural community located in the North East alpine area of Italy, in the region of Trentino-Alto Adige/Südtirol.

## Case Study Background

Our case study is a small municipal area of the Fiemme Valley, located in the province of Trento, in the north-eastern Italian Alps. This province is part of Trentino-Alto Adige/Südtirol Autonomous Region, and it represents one of the most interesting examples in the Italian energy efficiency scenario. According to a Green Economy Index recently reported by the Italian Chambers of Commerce (Unioncamere, 2018), Trentino-Alto Adige/Südtirol is the region that contributes most to the path of sustainable energy transition in Italy. The use of sustainable resources is in fact one of the milestones of the energy policy in the Trento and Bozen provinces that compose the autonomous region. The supplying of raw material in this area is mainly derived by wood chips from local wood industry and forestry. Forests cover more than 60% of the provincial surface area and represent a significant economic and environmental resource for the entire community, also in the perception of the inhabitants (e.g., Carrus et al., under review). Specifically, our case study is based on the Municipality of Cavalese (4,000 inhabitants). In the 2017 this small town was awarded a national “100% renewable municipality” prize, delivered by “Legambiente,” a major environmental NGO in Italy. The municipality of Cavalese began its path toward the energy transition in 1999, when the local political administration decided to build the first biomass district heating plant in the Trentino-Alto Adige/Südtirol region. This was an innovative experience in Italy and for the community, giving up methane to exploit the wood biomass derived from the Fiemme valley and from the neighboring Fassa valley. Woody biomass is exploited for the production of thermal energy and electricity in the area, and the heating plant of Cavalese is mainly provided by local sawmills (Buonocore et al., 2019). An accurate forest resource management, which characterizes the entire Trentino-Alto Adige/Südtirol region, guarantees the sustainable exploitation of local forest eco-systems through precise cutting practices, which allows forests to naturally regenerate over time. The long-term sustainability of timber production in the area, as well as the integrity of the forest ecosystems, is guaranteed by maintaining a balance between wood biomass increase and felling rates.

## Method and Participants

The case study approach is frequently used in social sciences as a research method for the comprehensive examination of a subject of study and its related contextual conditions, via in-depth data collection involving multiple sources of information, allowing to report a case description and case themes (Creswell, 2013). Our qualitative approach was based on the collection of in-depth semi-structured interviews with three different representatives of the Cavalese political, administrative and economic community, including a top-level administrative executive, a mid-level manager and an operational/field employee. By this, we intended to represent varying degrees of familiarity with the case study and to reinforce the robustness and solidity of our analysis. Interviewees were recruited through the involvement and support of the municipal administration, which helped us to identify the key-informants who best matched the purposes of our study. The added value of getting responses from expert key informants is also linked to this possibility of having access to an “insider” point of view about the sustainable transition process implemented by the formal social unit under our focus. Each of them tells in fact the story of the community, which is his own story.

The interviews were carried out following a semi-structured protocol. The interview guide is shown in Appendix A. Key sections aligned with the interview questions were related to the role of each stakeholder in the conception and development of the roadmap that lead to the start of the sustainable energy transition path, as well as to its practical implementation phase, its impact and its current outcomes. An informed consent form was signed by each interviewee. All the interviews were recorded and conducted in the native language of the stakeholders. To comply with ethical and privacy issues of Italy’s and the EU’s legislation, we decided to anonymize all participants, so that the interview parts reported here are attributed to interviewees and not to specific individuals; also, masculine personal pronouns are used throughout, regardless of the gender of the respondents. To enhance the epistemic meaning of our data collection, and to reduce the interviewer’s influence and the interviewee’s self-presentation bias, it was decided to conduct interviews in the presence of the three representatives simultaneously, as a sort of micro focus group. Although the participants were left free of intervening during each other’s interview, we do not analyze here the interactions between them. The reason why a group interview was conducted is twofold. On the one hand, the matter involves a social-psychological dimension, as it deals with issues pertaining to interpersonal choices and relationships; in this sense, the possibility of carrying out a comparison between three decision-making actors with respect to energy policies was certainly a methodological advantage. On the other hand, through the narrative of our key informants, we have an opportunity to better understand the specificity of the socio-cultural context they represent. In other words, this method registers and co-constructs a shared narrative in the cultural and local context of interaction. In this sense, the two logical and temporal dimensions that characterize the use of interviews in social-psychological research are encouraged: the “diachronic” one (which is specific to the in-depth analysis of each story told through the interviews) and the “synchronic” one (which allows to compare the contents transmitted by the different interviews on the same topic or content).

All the participants had an active role in the implementation of the district heating power station and in integrating the transition to sustainability in the decision-making processes, and in the administration of the local community. In particular:

1. the first interviewee is a top-level executive (#1_int) of the local administration; s/he played a decisive and important role in the promotion and maintenance of the energy transition process in the municipality area;
2. the second interviewee is a mid-level manager of a local energy provider company (#2_int); s/he has been working for long-time in the transition process from the very beginning of the energy transition process in the local area, as an expert in the renewable energy field throughout his professional career;
3. the third interviewee is an operational/field employee, who has worked as an employee in the Forestry Office for 40 years and is now retired (#3_int); when the project of district heating power station was initiated, s/he was involved also in the management of the forest and natural resources.

### Qualitative Data Analysis

The semi-structured group interview was subjected to qualitative content analysis through a coding procedure for “narrative themes” (Ryan and Russell Bernard, 2003; Braun and Clarke, 2006). Prior to the content analysis, every interview was transcribed in Italian and then translated in English; the answers from each participant were identified in order to obtain three interviews each relating to the individual participant.

Subsequently, in each of the interviews transcribed, we identified the themes proposed in the model by Steg et al. (2015), as described in the graphic representation displayed in Figure 1. In the qualitative approach tradition, a theme is important because it captures something meaningful about the data in relation to the research questions, and it represents patterned responses or meanings within the data set (Braun and Clarke, 2006, p. 82). We used the software MAXQDA for a computer-supported qualitative content analysis to filter out the structure of our transcripts and to code them (Kuckartz, 2013; Kuckartz and Rädiker, 2019).

In a first phase, three main thematic areas were primarily identified through the reading of the interviews, corresponding to the three main factors of sustainable energy transitions proposed by Steg et al. (2015; see Figure 1), and leading to the definition of the main content analysis categories:

(1) factors underlying energy saving behavior (i.e., factors on the basis of which the inhabitants of a community are more likely to engage in behaviors that promote the transition to renewable energy);
(2) interventions aimed to promote the transition toward renewable energy sources;
(3) public acceptability of social policies and changes in the energy production and distribution systems.

After identifying the interview parts pertaining to each of these thematic areas (“code sets” in the MAXQDA jargon), we proceeded to deepen the analysis by extrapolating the specific contents conveyed by text segments from the interview transcripts. Each interview segment in which there were present topics related to the three areas described above was (re)coded so as to more accurately detect the specific “narrative theme” around which the discourse of the interviewees is constructed; for each theme (“codes” and “sub-codes” in the MAXQDA jargon), the corresponding segment has been identified and defined. The “denser” segments (i.e., articulated, complex, polysemic) have been coded with more than one code or sub-code. To avoid confusion, it is important to recall that the terms code sets, codes and sub-codes belong to the MAXQDA jargon, and correspond to thematic areas (code sets) and themes (codes and sub-codes) in the narrative analysis terminology.

The results are described in the next section, and are organized around two different levels of connected analysis:

(1) the thematic area and the related narrative themes: what they are, which is their weight in quantitative terms (frequency of coded segments) and qualitative (which meaning they convey in the specific context in which they are elicited);
(2) the similarity and differences between the visions and the interpretations of the three different interviewees.

We also tried to understand if these thematic areas and themes are transversally connected to each other in narrative terms. That is, if in the course of the narratives produced with the interviews, the three participants simultaneously evoke themes related to different areas.

## Results

In this section we describe the emergence of thematic areas (code sets) and themes (codes and subcodes), and their relations, in order to clarify the conceptual frame emerging from the data.

A specific content analysis is provided and presented for of each of the main theme identified and described above: (1) factors underlying energy saving behavior; (2) interventions to promote the renewable energy transition; (3) public acceptability of social policies.

### Factors Underlying Energy Saving Behavior

**FIGURE 2:**
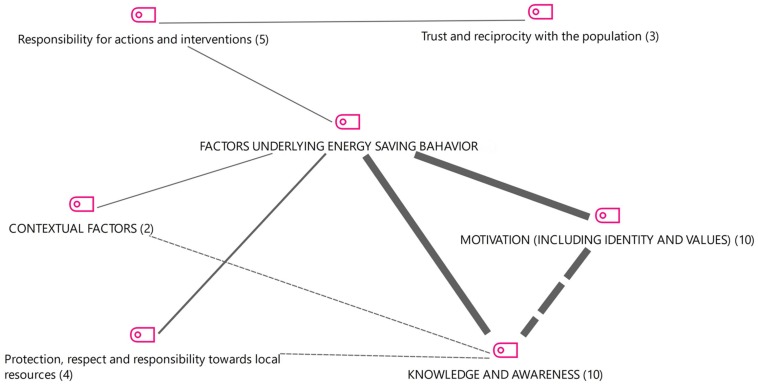
Factors underlying energy saving behavior: overall picture as emerged from the qualitative content analysis.

Our analysis started from the thematic area “Factors underlying energy saving behavior” proposed by Steg et al. (2015). In Figure 2, the main themes that are present in Steg et al. (2015) are reported in capital letters, while the additional themes we found in our interviews as grounded in the specific experience of our key informants are in reported in lowercase.

As Figure 2 shows, participants’ discourses refer to the responsibility for actions and interventions, trust and reciprocity with the population, and protection, respect and responsibility toward local resources. These themes recall a strong rooting in the territory and in the community. Moreover, the arguments developed here have a solid relational meaning (reciprocity, responsibility, respect, trust). Compared to the Steg et al. (2015), which mostly outlined individual-based factors nature, our analysis also identified more “social” factors underlying the pro-environmental behavior, which are rooted into the fundamental principles of a small community life.

It seems that psycho-social themes play a role as motivational factors too. The maintenance of a sense of identity and the sharing of values both work as a motivational incentive, because local inhabitants develop an “awareness of the value of one’s assets, of one’s own territory” (#3_int). We guess that this sense of belonging stands out, and it is understood by our key informants as a manifestation of one’s own identity and of the values connecting people each other. A strong bond seem to emerge between the institutions and the population: we find this theme when interviewees talk about “trust” and “responsibility”: “broadly speaking, if we can also be grateful to the territory (to be intended both as an environment and as population), this is something that allows to establish and strengthen a relationship of mutual trust, which is fundamental for the development of new initiatives” (#2_int).

Through the awareness of the local identity and values, contextual factors (to be intended as environmental and social constrains) have an impact of energy saving behaviors. This is a characteristic recognized in the inhabitants of Cavalese, which are presented to show an “intrinsic fact in the culture of the people from the mountain, that is the fact of being attached to their territory, of having a very positive idea of own land” (#2_int). This place attachment feeling seem to have pushed the community toward a conscious and sustainable choice: “the widespread sensitivity in this direction has favored this development process” (#2_int). From a specifically social and environmental psychological point of view, the attachment to the territory and the place identity are recognized as the main motivators that push and support the choice of moving toward a more sustainable energy system. But other more concrete motivators can also be identified: “in addition to solidarity and the cultural one, there is also a personal economic return” (#1_int). This material motive is linked to the potential monetary savings of a new heating system, compared to a traditional one, so that “in the early times there have been restitutions in the form of energy, now there are refunds, even in economic form for the small shareholder” (#1_int).

**FIGURE 3:**
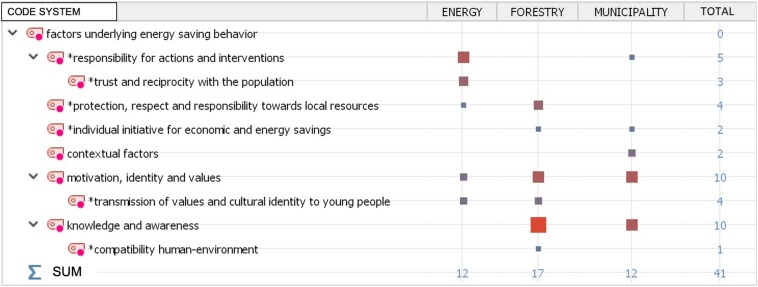
Factors underlying energy saving behavior across the different key informants.

Moving to a further level of analysis, we aimed to compare the visions of the three interviewees. The MAXQDA software extrapolates the number of coded segments for each interviewee and compares them. This process produces a hierarchy of themes (which are displayed in the rows of Figure 3) for each interviewee (which are displayed in the columns of Figure 3). At the intersection between the rows and the columns, the size and color of the squares indicates the frequency of the segments encoded for each interview. The marginal row and column totals are provided for a more quantitative reading of the output. Here, again, for all three respondents, we can highlight the importance of issues of local and cultural identity, which is presented as a fundamental aspect for the successful outcome of the energy transition (see Figure 3).

While recognizing this aspect as fundamental, an extract from the #1_int also highlights the important role played by institutions in this process: “there is a plenty of complex factors that can be developed and put in place by the individual citizen, but these information must be supported by institutions; therefore (there is) a synergy, in such a way that everyone feels part of a community and it is also responsible for the sustainability of what the community does.”

### Interventions to Promote the Renewable Energy Transition

**FIGURE 4:**
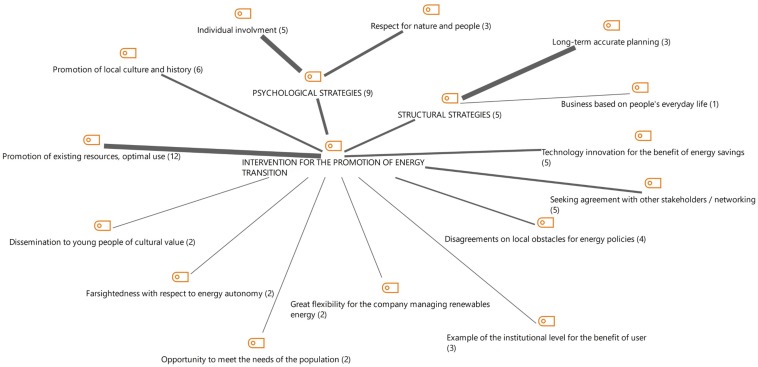
Interventions to promote the transition toward renewable energy sources: overall picture as emerged from the qualitative content analysis.

**FIGURE 5:**
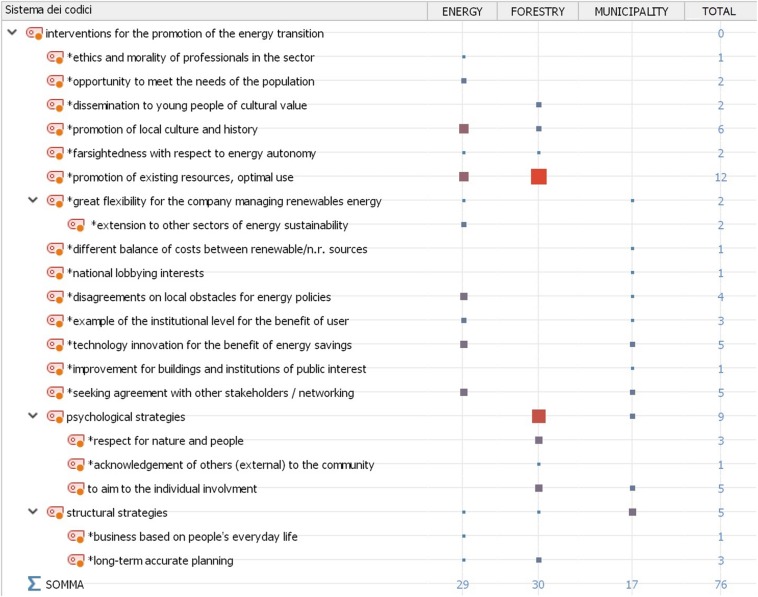
Interventions to promote the transition toward renewable energy sources across the different key informants.

We then focused on the thematic area of “Interventions in favor of promoting the transition toward forms of energy from renewable sources.” Here, there seems to be a strong emphasis on concepts related to what Steg et al. (2015) have defined as “psychological” strategies (see Figures 4, 5). The following quote is representative of this kind of narrative arguments, where the setting up and implementation of concrete projects is linked to the issue of respect and value for nature and people:

“(#1_int): The answer is not so powerful, and this also depends somewhat on the abilities or perhaps on the cultural substratum of each of us. There is no doubt that the sensitivity to these universal problems varies across individual; primarily, the task of the institution is precisely to try to involve the individual to let people adopt sustainability behavior relying on his own responsibility.”

“(#3_int): we focused on Mother nature and on a use that has always been correct, even when there were no national or local rules, absolutely. To save our resources, we must use them within certain limits and with certain criteria. And this is something, I would say - a big word - it’s in the DNA of these people.”

Reference to the so-called “structural” strategies is also present, albeit with less emphasis, as illustrated by the following quotes, where the interviewees point out the importance of a long-term careful planning in the context of the political and social specificities of the Fiemme Valley:

“(#2_int): the topic is much broader, we tried to develop local and territorial initiatives within a logic of valorization of waste. And therefore this heart, this principle that inspires our action is applicable and can be applied to many sectors: in the wood supply chain on which Cavalese has invested, but also for example in the chains of differentiated collections, of the collection of the organic fraction on which we have developed other entrepreneurial initiatives that however start from the same matrix. And from a whole series of other initiatives we are developing.”

“(#3_int): What are the objectives? From here there are also indications, in principle clearly, on how to work to achieve those objectives. But then planning must be translated into reality, because if you stay around a table, we have anything. And here is where the planner must become an operator.”

From her/his specific point of view, the interviewee who has the stronger administrative and political background, explicitly recognizes the role of institutions in supporting sustainable behavior, as illustrated in the following extract: “(#1_int): In local terms, energy policies, the provincial energy plan is very advanced from this point of view, it is very much moved toward sustainability and use of renewable resources”; but in her/his view, institutions are also responsible for some of the main barriers encountered, especially with regard to the administration times, which do not allow the easy implementation of the projects: “(#1_int): If the scenario is national or global, things absolutely change”; “(#1_int): We are slow, very slow, from an administrative point of view in carrying out procedures and this happens regularly in public works. So, we run the risk of making a project and seeing it realized when things are already old.”

A similar barrier is mentioned by the #2_int: “the bureaucracy understood as an obstacle to development, but also certainty of the norm […] the state that overnight changes these rules or perhaps interprets them through the state offices, […] Consequences that there are in terms of times of clarifications, times of interpretations, times of justice. this is what produces the removal of investments and companies: the uncertainty, in addition to the bureaucratic difficulty.”

The relationship between national and local regulations causes a debate between two interviewees. In one case, #2_int believes that the relationship with national legislation has slowed down the local autonomy, and consequently the local energy transition:

(#2 int): “we live in a world where today everything is very complicated, with reaction times that are too long, within a system – the energy one, because we do this too, in addition to culture and communication, etc. – which is national, which in fact suffers national rules, although here it is an autonomous province, which has reaction times compared to the speed of the world that are incompatible, are absolutely incompatible.”

However, #1int believes that the transition project has gone ahead independently of the bureaucratic obstacles and national regulations:

(#1_int): “if we reason in local terms, I cannot see any barriers, because energy policies and the local energy plan are very advanced, it is much shifted toward sustainability and the use of renewable resources.”

Both these segments can be coded as “disagreements on local obstacles for energy policies.” Related to this, the #2_int cites another factor that could represent a barrier: cultural homologation, exemplified by reference to terms such as “identity aspects, autonomous matrices, territorial values, risk being overwhelmed and swept away.” This issue is shared also by the other interviewees, for example through the fear that “the new generations lose the cultural identity, the foundations, the roots that have been at the base of these results” (#3_int).

As for the distribution of these thematic areas and themes in the three interviews, Figure 5 shows that the #3_int proposes some themes, which are also closely related to her/his role, and which are only partially shared by the other interviewees, such as the “enhancement and optimal use of resources” (a common theme also for the interviewee who holds a mid-level manager position), the “respect for nature and people” and “the involvement of the individual.” While the last two aspects are mentioned by Steg et al. (2015) as an integral part of psychological strategies, the theme of “enhancement and optimal use of resources,” is not immediately traceable in their general framework. The following segments might help to clarify the meaning of this theme that emerged from the interviews:

(#2_int): The topic is much broader, that is to say, to develop local and territorial initiatives within a logic of valorization of waste. And, therefore, this core principle inspires our action and it can be applied to many sectors: in the wood supply chain on which Cavalese has invested, but also for example in the chains of differentiated collections, of the collection of the organic waste on which we have developed other entrepreneurial initiatives. And from a whole series of other initiatives we are developing.

(#3_int): The same thing - let me tell you - also concerns all the other resources of the territory as a whole. For example, starting in the 1960s there were strong requests for tourist development, runways, ski lifts and so on. The same applies to hydroelectric power, it is very good because even water is a renewable raw material, but water is a biological quantity, a naturalistic greatness in the widest sense.

(#3_int): I tell you for example about the forest, every minute that passes from the 60s we have two more square meters of forest. Every three minutes we have one more cubic meter of wood, of wood mass. Here I speak about the entire Fiemme Valley, that is 150.000 cubic meters per year. If 100.000 are used, 50.000 are saved so that this resource can always be in optimal conditions. But it is not only a question of quantity, but also of quality of action. And, I mean, you don’t take a tree except to grow many more. And that’s why when you look around, many say: but, do you never cut a tree here? No, it’s not true, absolutely. Men and nature can have equal dreams.

In addition to that, we can observe also a strong association of these themes to issues pertaining to local policies and the sense of community, which is a concept not so explicitly present in the general model we are referring to. This emphasis on the localization of the possible interventions is also corroborated by the fact that the most frequent themes are the promotion of existing resources for their optimal use (12 coded segments, shown on the left side of Figure 4) and the promotion of local culture and history (6 segments). From the exploration of these themes it thus emerges the idea of a community well aware and conscious of sustainable choices: “the widespread sensitivity in this direction has favored this development process” (#2_int). Indeed, a sense of community emerges, and it guarantees people that they can “do together” within a trustworthy relationship: “there is trust in our territory, trust in people.” (#2_int). These founding feelings of the Cavalese community are also what allowed the “our company to exist, since individuals have had a fundamental involvement in the birth, first of all it is also – in addition to being a customer […] – it is also a shareholder” (#2_int). The community is therefore directly involved in energy choices “it is a public company where no one, neither public nor private, has absolute control and where, however, all parts of the territory have a say finding a way of business management” (#2_int).

Our participants insist thus on the need to keep cultural values and respect for the identity of people together. It is possible to affirm that the psychological strategies, according to the vision of our interviewees, have played (and still play) an important role in continuing the process of energy transition started over the years. This seems to prevail especially in the vision of the (#3_int), as illustrated in the following extracts:

(#3_int): Indeed, it is convenient. I add – so, because we have to say also for psychology – which helps a little bit… The appreciation coming from outside. People say: ‘look at the beautiful environment that you have, as it is clean, look at the beautiful woods you have.

(#3_int): The results. In the sense, the goal is something you want to achieve, but when you see that you reach them, you say: but look a bit, maybe it was worth it. And look, they are not just environmental objectives, because we must never forget man. Things are always joined together. When I saw some distrust in my co-workers and others in bringing forward ideas, be present, look, and then when someone told me: but do you know that it is true journey… And so talk with the environment, with the territory, but talk to people.”

### Public Acceptability of Social Policies

**FIGURE 6:**
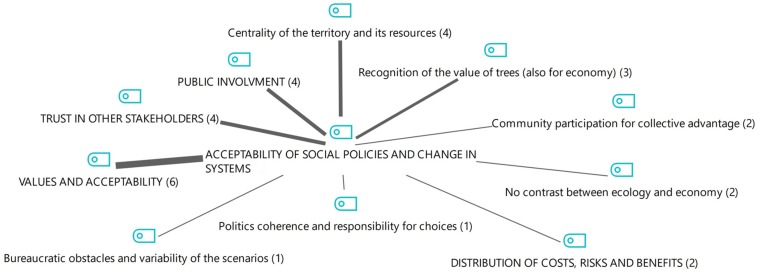
Public acceptability of social policies and changes in the energy domain: overall picture as emerged from the qualitative content analysis.

**FIGURE 7:**
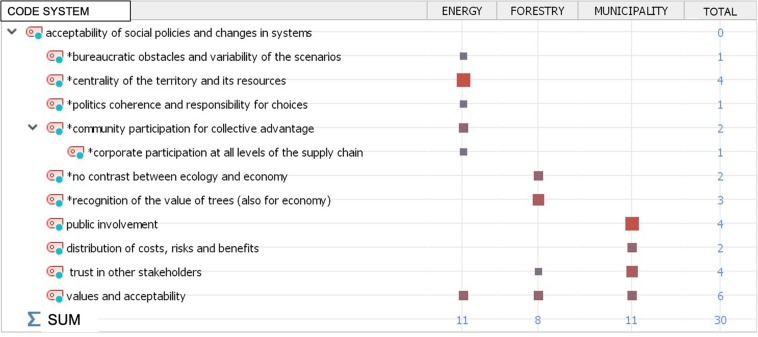
Public acceptability of social policies and changes in the energy domain across the different key informants.

Finally, our analysis referred to the thematic area of the “Acceptability of social policies” and changes in systems. Here, it is worth to underline how the interest related to the methods of accepting social policies centered on the use of renewable energies is a common theme for all interviewees. Specifically, the concept of values and their acceptability in the community is one of the most common themes in all interviews (see Figure 6), shared by all respondents (see Figure 7).

Two issues that are particularly relevant in a social psychological perspective emerge: trust from/to other stakeholders in the transition process and the need for public involvement through a direct relation with the community. This point can be exemplified by the points of view of different interviewees, as in the following extracts:

(#3_int): “we need to restore more trustful relationships between people. Few rules but certain, but clear. Everything that is said and done must be understandable, it must reach the heart of the problems. It must reach people without too many intermediaries. And you think there were these things here…”.

(#1_int): “But already years before this path had begun, therefore designing the networks, asking for adhesion to small shareholders, to the sawmills of the valley, to the rural bank. And the journey began from there.”

As mentioned above, the acceptability of the energy transition values is a theme shared by the three interviewees, as illustrated also by the extracts we report below:

(#2_int): “the population is naturally more willing to follow – perhaps even with some complaints, with some mutterings, because they are mountain people but it is also people who when they complain and when they get angry make you feel – but always within a context, within a relationship of trust. Here, trust – in addition to participation and what we said, so a whole series of elements that are on the territory - but trust is the other part of the coin. In the term “trust” is summed up a bit, what you say: there is trust in our territory, trust also in people. this allowed in short and still allows even if here some small crack is beginning to be seen.”

(#3 int): “This path has also begun to bring something innovative to the valley. We are a valley rich in raw materials, above all wood, and there was this possibility to be exploited in an intelligent way, namely that of using the residues of the use of the timber that was made in the valley, and that is still made, to produce energy and therefore close what is an energy balance, a circular economy.”

## Discussion and Conclusion

The use of qualitative techniques allows for deep insights in understanding human experience in relation to energy decisions and decision-making processes in collective settings, particularly during low carbon energy transition processes (Strauss and Corbin, 1990; Kvale, 1996). The aim of using interviews in this paper was to discover common and emerging themes, distinctive aspects, patterns, sequences and causal relationships, possible hierarchies, and the general context of decision-making processes in collective units, as reported directly by the narrative of insider players in the decision-making bodies. It utilizes a narrative process of interpretation that is useful in gaining a deeper understanding of the drivers behind collective energy choices. In our study, we focused in particular on the point of view of formal decision makers involved in the implementation of an energy transition process. As known, it is commonly accepted that the top-down dynamics in the decision-making process are crucial in the energy domain. However, our analysis also revealed the existence of some more bottom–up dynamics, clarifying the interaction process among different levels of environmental decision-making. Consequently, bottom–up dynamics also resulted as a noteworthy driving force for obtaining the positive outcomes and the successful implementation in the energy transition project we studied.

The case study described in this paper is particularly interesting, because, according to the voices of the key informants, its success and positive outcomes were the product of a synergic and productive collaboration between different levels of authority and governance, which necessarily had to take into account the different interests and needs of the stakeholders present in the territory. One of the aspects that emerges immediately from our qualitative work is the presence and strengthening of positive relations between local authorities, stakeholders, and citizens. In particular, the attention to the participation of citizens in the decision-making processes, and to importance assigned to the implementation of transition actions based on the enhancement and respect of the natural resources of the territory, is reported consistently by our respondents, and framed in their discourse as a key element for the successful outcomes of the sustainable transition.

Among the main factors underlying energy saving behavior that characterized the process of energy self-sufficiency initiated by the formal social unit described in this case study, the importance of collective identity and self-definition processes emerged as prominent in the dynamics of community’s pro-environmental behavior, as presented by the decision makers involved in our study. Specifically, place attachment and local identity are recognized as the main motivators which currently drive and support the choice of sustainable energy as a shared vision of the community. The salience of a collective identity dimension that emerges from the qualitative analysis is suggestive of how social and environmental responsibilities are considered as intertwined for the development of good ecological practices in the vision of the representatives of the community we focused on. Consequently, the motivation to defend and promote collective identity in the local territory seems to have stimulated a greater attention and responsibility toward the environment.

In addition, our qualitative approach shows how a strong place attachment allows to develop an awareness of the value of their territory, among the local inhabitants. This aspect, which is coherent with many previous studies on place attachment and pro-environmental behavior (e.g., Scannell and Gifford, 2010; Carrus et al., 2013) is intimately recognized among the decision makers and, according to them, among community’s residents as well. In the view of our respondents, it represents the main factor that steered all the stakeholders toward the conscious adoption of a more sustainable lifestyle. Our key informants suggested that it was fundamental to work on the values and sense of social identity of the community, as in their experience this has influenced personal choices fostering those cooperative processes that are at the basis of ecologically oriented human behavior. Based on the narrative themes we identified, it is arguable that the acquisition of a stronger environmental awareness has contributed to implementing gradual behavioral changes in the community, leading to a significant transformation of citizens’ lifestyles over time. As pointed out by previous authors, a strong place attachment and sense of community allows people to feel as an active part of the decision-making process, being at the center of the political actions toward environmental sustainability and the use of renewable energy sources (e.g., Hoffman and High-Pippert, 2010; Dixon et al., 2015). The sense of community is linked to participation in public life and the sharing of activities, interests and values about the human-environment relationship. Citizen’s participation in community policy making is extremely relevant for effective political and social interventions (e.g., Churchman and Sadan, 2004). Citizen involvement in decision-making and operational processes might implement the sense of community and the salience of social identities and promote willingness to take part in sustainable initiatives (e.g., Bamberg et al., 2015). Previous research indeed identified sense of community as a key factor in sustainable transitions, as it promotes a relationship based on trust between local authorities, stakeholders, and citizens (e.g., Van Vugt, 2002; Carrus et al., 2013). The importance of social identification processes emerges from the overall narrative presented by our respondents, who consistently point out that local identity could have been a key factor to promote cooperation between citizens and authorities, by developing bonds based on trust and on social and environmental responsibility. Again, this is strongly consistent with previous theories and empirical findings on pro-environmental behaviors (e.g., Fritsche et al., 2018).

In our overall approach, we started from the exhaustive review of the human dimension of sustainable energy transitions provided by Steg et al. (2015). Our data seem to give further support to this proposal, also when exploring directly the point of view of key representatives of formal social units, and not just the attitudes, beliefs and cognitions of single individuals. Looking in particular at the connections between the different narrative themes relating to the factors underlying sustainable behaviors, as emerging from our qualitative analysis, a strong reference to specific psychological aspects such as motivation and affect is clearly present.

Our interpretation of the data described above reveals the presence of a strong sense of community as a specific dimension of participation in community life that includes belonging and the motivations connected to the exercise of social action and responsibility. In the view of our respondents, this has been a fundamental aspect to ensure the success of the local sustainable energy transition (see also Van Der Schoor and Scholtens, 2015). Our analysis also highlights how the sense of responsibility plays a central role in the discussion on environmental competences and on the possibility of intervening effectively on citizens’ behaviors and consumption styles. Feeling responsible for the natural resources available in one’s living environments might allow for a better management of such resources, and might boost the caring of the natural environment in the community.

As mentioned earlier in this paper, the Cavalese town and Fiemme valley community is characterized by a strong sense of local identity that has developed over the centuries, and this permeates people’s daily lives, as it often happens in the Alps and other mountain areas, or in other peculiar geographical contexts. Here, the local administration, the companies and the territorial institutions have planned and launched interventions aimed at enhancing this identity component among the population, with the explicit vision of promoting the public acceptance and thus the long-term success of the transition process.

Thus, among the psychological strategies mentioned by Steg et al. (2015) as a fundamental mechanism for achieving the sustainable energy transition, beyond the strictly individual factors like attitudes, beliefs or values, we can argue for the inclusion of more social factors such as the institutional respect for people and organized groups, for their identity and their local culture. Any proposal for change and innovation that formal social units plan to implement should thus not be made in an abstract social vacuum, but rather tailored to context specificities, for example through the enhancement of existing natural resources and social capital. At the same time, the direct involvement of people in the territories favors a greater and more authentic motivation for change, especially if combined with a sense of local and cultural identity which is more likely to be perceived as consonant with the values of the community (e.g., Carrus et al., 2005). According to the local decision makers, the involvement and participation of citizens in the community governance system and the role and functions of political representatives and stakeholders was integrated into a comprehensive vision of the social processes, that guided the action of the various actors engaged in the local energy transition.

To conclude, the results of our case study suggest how the knowledge of the processes and the awareness of being at the center of the change facilitated the community endorsement of the transition process, through the promotion of a sense of direct responsibility for both the development of the territory and the protection of the environment therein.

## Data Availability Statement

The datasets generated for this study are available on request to the corresponding author.

## Ethics Statement

Ethical review and approval was not required for the study on human participants in accordance with the local legislation and institutional requirements. The patients/participants provided their written informed consent to participate in this study.

## Author Contributions

LT contributed to conception and design of the manuscript, undertook fieldwork taking contact with the three representatives and conducting the interviews, and wrote a first draft of the manuscript. ED performed the analysis and wrote the results sections. GC revised the first draft of the introduction and results, wrote the remaining parts of the manuscript, and edited the final version. MB and MD conceptualized the research in ECHOES work package and wrote part of the introduction of the manuscript. ED, LT, and GC interpreted the results and jointly wrote the discussion section. AP contributed to manuscript revision. All authors have read and approved the final manuscript.

## Conflict of Interest

The authors declare that the research was conducted in the absence of any commercial or financial relationships that could be construed as a potential conflict of interest.

## Footnotes

1 https://www.irena.org/energytransition

2 https://ec.europa.eu/energy/en/topics/energy-strategy/energy-union-0

3 https://ec.europa.eu/energy/en/topics/technology-and-innovation/strategic-energy-technology-plan

4 https://echoes-project.eu/

## Appendix

The following section contains the key sections and the semi-structured interview questions for surveying the psychological and social factors affecting the energy choices and behavior of a formal social unit in the specific case study considered, and how these contributed to foster a collaborative action and to manage the social, cultural, and socioeconomic dynamics in the energy transition path of the involved community.

### (1) Description of the Actual Problem/Idea/Case/Project:

(a) Please tell me briefly about your organization and your background.
(b) How long have you been with your current organization?
(c) What is your role in the organization?
(e) Please describe the Actual Problem/Idea/Case/Project briefly.
(f) Please provide us with an historical overview of the events and context that led to seek for a solution for the Actual Problem/Idea/Case/Project.

### (2) Analysis of the Existing Alternatives

(a) Nowadays, there is a great deal of attention to the low-carbon energy transition. You as a top-level executive/mid-level managers/operational/field employee, how do you perceive low-carbon energy transition and how do you consider the importance of low-carbon energy transition with relevance to your Actual Problem/Idea/Case/Project?
(b) What alternatives were present regarding the Actual Problem/Idea/Case/Project?
(c) How did the organization select among alternative solutions? Briefly mention the top-down and bottom–up dynamics in decision-making process.

### (3) Development of the Road Map and the Solution Approach

(a) How do you consider the rationale behind selecting a solution to the case considering the decision-making process?
(b) How was the case/project kick-off decided in the organization?
(c) How can you identify the planning steps that were followed?
(d) Please briefly describe the monitoring and tracking methods implemented for the roadmap.

### (4) Implementation Phases

(a) You as a top-level executive/mid-level managers/operational/field employee, how do you see your role-play in the implementation of the solution to the Actual Problem/Idea/Case/Project?
(b) Please briefly describe how the solution to the case was implemented.
(c) What were the challenges encountered during the implementation phase?
(d) How would you identify the barriers and motivators that were effective during the implementation?

### (5) Results of the Implementation

(a) You as a top-level executive/mid-level managers/operational/field employee, how would you rate the success of the solution and why?
(b) You as a top-level executive/mid-level managers/operational/field employee, do you think the expected results were accomplished? Why/why not?
(c) You as a top-level executive/mid-level managers/operational/field employees, can you evaluate the results based on the key stakeholders’ perspective?
(d) How was the effectiveness of the solution measured?
(e) What factors played a key role in the effectiveness of the solution?

### (6) Impact and Diffusion of Results

(a) You as a top-level executive/mid-level managers/operational/field employees, how do you see the long term effects associated with the case including the impacts on the organization, its environment and stakeholders?
(b) Where is this association headed in the future with the experience generated from the Actual Problem/Idea/Case/Project?

## References

[B1] Abrahamse W. Steg L. Vlek C. Rothengatter J. A. (2005). A review of intervention studies aimed at household energy conservation. J. Environ. Psychol. 25 273–291.

[B2] Bain P. G. Kroonenberg P. M. Johansson L. O. Milfont T. L. Crimston C. R. Kurz T. (2019). Public views of the sustainable development goals across countries. Nat. Sustain. 2 819–825. 10.1038/s41893-019-0365-4

[B3] Bamberg S. Rees J. Seebauer S. (2015). Collective climate action: determinants of participation intention in community-based pro-environmental initiatives. J. Environ. Psychol. 43 155–165.

[B4] Biresselioglu M. E. Demir M. H. Demirbag Kaplan M. Solak B. (2020). Individuals, collectives, and energy transition: analysing the motivators and barriers of European decarbonisation. Energy Res. Soc. Sci. 66:101493 10.1016/j.erss.2020.101493

[B5] Braun V. Clarke V. (2006). Using thematic analysis in psychology. Qual. Res. Psychol. 3 77–101. 10.1191/1478088706qp063oa 32100154

[B6] Brondi S. Armenti A. Cottone P. Mazzara B. M. Sarrica M. (2014). Parliamentary and press discourses on sustainable energy in Italy: no more hard paths, not yet soft paths. Energy Res. Soc. Sci. 2 38–48.

[B7] Brondi S. Sarrica M. Caramis A. Piccolo C. Mazzara B. M. (2016). Italian parliamentary debates on energy sustainability: how argumentative ‘short-circuits’ affect public engagement. Public Understand. Sci. 25 737–753. 10.1177/0963662515580067 25904600

[B8] Buonocore E. Paletto A. Russo G. F. Franzese P. P. (2019). Indicators of environmental performance to assess wood-based bioenergy production: a case study in Northern Italy. J. Clean. Product. 221 242–248.

[B9] Carrus G. Bonaiuto M. Bonnes M. (2005). Environmental concern, regional identity, and support for protected areas in Italy. Environ. Behav. 37 237–257.

[B10] Carrus G. Chokrai P. Fritsche I. Klöckner C. A. Masson T. Panno A. (2019). Psychological factors in energy decisions: Results from experimental studies and a multinational survey. ECHOES report no. D4.2 (H2020 project no. 727470). Available on https://echoes-project.eu/sites/echoes.drupal.pulsartecnalia.com/files/D4.2%20%28R1%29.pdf (accessed December 31, 2019).

[B11] Carrus G. Panno A. Leone L. (2018). The moderating role of interest in politics on the relations between conservative political orientation and denial of climate change. *Soc. Nat. Res.* 31 1103–1117. 10.1080/08941920.2018.1463422

[B12] Carrus G. Scopelliti M. Fornara F. Bonnes M. Bonaiuto M. (2013). “Place attachment, community identification, and pro-environmental engagement,” in Place Attachment: Advances in Theory, Methods and Applications, eds Manzo L. Devine-Wright P. (New York: Routledge), 154–164.

[B13] Churchman A. Sadan E. (2004). “Public participation in environmental design and planning,” in Encyclopedia of Applied Psychology, ed. Spielberger C. (Oxford: Elsevier), 793–800.

[B14] Creswell J. W. (2013). Qualitative Inquiry and Research Design: Choosing Among Five Approaches. Thousand Oaks, CA: Sage.

[B15] Dixon G. N. Deline M. B. McComas K. Chambliss L. Hoffmann M. (2015). Saving energy at the workplace: the salience of behavioral antecedents and sense of community. Energy Res. Soc. Sci. 6 121–127.

[B16] Dumitru A. De Gregorio E. Bonnes M. Bonaiuto M. Carrus G. Garcia-Mira R. (2016). Low carbon energy behaviors in the workplace: a qualitative study in Italy and Spain. Energy Res. Soc. Sci. 13 49–59.

[B17] European Climate Foundation (2010). Roadmap 2050: A practical guide to a prosperous, low-carbon Europe. Available at https://www.roadmap2050.eu/attachments/files/Volume2_Policy.pdf (accessed December 31, 2019).

[B18] Evans G. W. Otto S. Kaiser F. G. (2018). Childhood origins of young adult environmental behavior. Psychol. Sci. 29 679–687. 29447064 10.1177/0956797617741894

[B19] Fritsche I. Barth M. Jugert P. Masson T. Reese G. (2018). A social identity model of pro-environmental action (SIMPEA). Psychol. Rev. 125:245. 10.1037/rev0000090 29265852

[B20] Geels F. W. (2010). Ontologies, socio-technical transitions (to sustainability), and the multi-level perspective. Res. Policy 39 495–510.

[B21] Geels F. W. (2014). Regime resistance against low-carbon transitions: introducing politics and power into the multi-level perspective. Theory Cult. Soc. 31 21–40.

[B22] Hirsh R. F. Jones C. F. (2014). History’s contributions to energy research and policy. Energy Res. Soc. Sci. 1 106–111. 10.1016/j.erss.2014.02.010

[B23] Hoffman S. M. High-Pippert A. (2010). From private lives to collective action: recruitment and participation incentives for a community energy program. Energy Policy 38 7567–7574.

[B24] Kaiser F. Arnold O. Otto S. (2014). Attitudes and defaults save lives and protect the environment jointly and compensatorily: understanding the behavioral efficacy of nudges and other structural interventions. Behav. Sci. 4 202–212. 25379277 10.3390/bs4030202 PMC4219263

[B25] Klöckner C. A. (2013). A comprehensive model of the psychology of environmental behaviour—A meta-analysis. Glob. Environ. Change 23 1028–1038.

[B26] Kuckartz U. (2013). Qualitative Text Analysis: A Guide to Methods, Practice and Using Software. London: Sage.

[B27] Kuckartz U. Rädiker S. (2019). Analyzing Qualitative Data with MAXQDA. Berlin: Springer.

[B28] Kvale S. (1996). Interviews: An Introduction to Qualitative Research Interviewing. Thousand Oaks: Sage.

[B29] Masson T. Panno A. Tiberio L. Vesely S. Carrus G. Fritsche I. (2017). Identity processes and individual factors in energy decisions: two comprehensive meta-analyses. ECHOES report no. D4.1 (EU H2020 project no. 727470). Available on https://echoes-project.eu/sites/echoes.drupal.pulsartecnalia.com/files/D4.1.pdf (accessed December 31, 2019).

[B30] Panno A. Carrus G. Leone L. (2019). Attitudes towards trump policies and climate change: The key roles of aversion to wealth redistribution and political interest. *J. Soc. Issues* 75 153–168. 10.1111/josi.12318

[B31] Ruepert A. Keizer K. Steg L. Maricchiolo F. Carrus G. Dumitru A. (2016). Environmental considerations in the organizational context: a pathway to pro-environmental behaviour at work. Energy Res. Soc. Sci. 17 59–70. 10.1016/j.erss.2016.04.004

[B32] Ryan G. V. Russell Bernard H. (2003). Techniques to identify themes. Field Methods 15 85–109. 10.1177/1525822X02239569

[B33] Sarrica M. Biddau F. Brondi S. Cottone P. Mazzara B. M. (2018a). A multi-scale examination of public discourse on energy sustainability in Italy: empirical evidence and policy implications. Energy Policy 114 444–454.

[B34] Sarrica M. Brondi S. Cottone P. (2014). Italian views on sustainable energy: trends in the representations of energy, energy system, and user, 2009–2011. Nat. Cult. 9 122–145.

[B35] Sarrica M. Brondi S. Cottone P. Mazzara B. M. (2016b). One, no one, one hundred thousand energy transitions in Europe: the quest for a cultural approach. Energy Res. Soc. Sci. 13 1–14.

[B36] Sarrica M. Brondi S. Piccolo C. Mazzara B. M. (2016a). Environmental consciousness and sustainable energy policies: Italian parliamentary debates in the years 2009–2012. Soc. Nat. Resour. 29 932–947.

[B37] Sarrica M. Richter M. Thomas S. Graham I. Mazzara B. M. (2018b). Social approaches to energy transition cases in rural Italy, Indonesia and Australia: iterative methodologies and participatory epistemologies. Energy Res. Soc. Sci. 45 287–296.

[B38] Scannell L. Gifford R. (2010). The relations between natural and civic place attachment and pro-environmental behavior. J. Environ. Psychol. 30 289–297.

[B39] Schultz P. W. Estrada M. Schmitt J. Sokoloski R. Silva-Send N. (2015). Using in-home displays to provide smart meter feedback about household electricity consumption: a randomized control trial comparing kilowatts, cost, and social norms. Energy 90 351–358.

[B40] Smil V. (2010). Energy Transitions. History, Requirements, Prospects. Santa Barbara, CA: Praeger.

[B41] Solomon B. D. Krishna K. (2011). The coming sustainable energy transition: history, strategies, and outlook. Energy Policy 39 7422–7431.

[B42] Sousa Lourenço J. Ciriolo E. Rafael Almeida S. Troussard X. (2016). Behavioural insights applied to policy: European Report 2016. EUR 27726 EN. Ispra: Joint Research Centre, European Union.

[B43] Steg L. (2008). Promoting household energy conservation. Energy Policy 36 4449–4453.

[B44] Steg L. (2016). Values, norms, and intrinsic motivation to act proenvironmentally. Annu. Rev. Environ. Resour. 41 277–292.

[B45] Steg L. Perlaviciute G. van der Werff E. (2015). Understanding the human dimensions of a sustainable energy transition. Front. Psychol. 6:805. 10.3389/fpsyg.2015.00805 26136705 PMC4469815

[B46] Strauss A. Corbin J. (1990). Basics of Qualitative Research Techniques and Procedures for Developing Grounded Theory. London: Sage Publications.

[B47] Thomas S. Richter M. Lestari W. Prabawaningtyas S. Anggoro Y. Kuntoadji I. (2018). Transdisciplinary research methods in community energy development and governance in Indonesia: insights for sustainability science. Energy Res. Soc. Sci. 45 184–194. 10.1016/j.erss.2018.06.021

[B48] Unioncamere (2018). Indice Green Economy: Spazio Per Tutti. Available online at: http://www.unioncamere.gov.it/csr/P42A1595C154S153/Indice-Green-%20Economy–spazio-per-tutti.htm (accessed December 31, 2019).

[B49] Van Der Schoor T. Scholtens B. (2015). Power to the people: local community initiatives and the transition to sustainable energy. Renew. Sustain. Energy Rev. 43 666–675.

[B50] Van Vugt M. (2002). Central, individual, or collective control? Social dilemma strategies for natural resource management. Behav. Sci. 45 783–800.

[B51] Verbong G. Geels F. (2007). The ongoing energy transition: lessons from a socio-technical, multi-level analysis of the Dutch electricity system (1960–2004). *Energy policy* 35 1025–1037. 10.1016/j.enpol.2006.02.010

